# Self-treatment of acute exacerbations of chronic obstructive pulmonary disease requires more than symptom recognition – a qualitative study of COPD patients’ perspectives on self-treatment

**DOI:** 10.1186/s12875-017-0582-8

**Published:** 2017-01-25

**Authors:** Johanna Laue, Hasse Melbye, Mette Bech Risør

**Affiliations:** General Practice Research Unit, UiT The Arctic University of Norway, 9037 Tromsø, Norway

**Keywords:** COPD, Exacerbations, Self-treatment, Patient perspective, Primary care

## Abstract

**Background:**

Self-treatment of acute exacerbations of COPD with antibiotics and/or oral corticosteroids has emerged as a promising strategy to reduce hospitalization rates, mortality and health costs. However, for reasons little understood, the effect of self-treatment, particularly when not part of comprehensive self-management programs, remains unclear. Therefore, this study aims to get insight into the patients’ perspective on self-treatment of acute exacerbations of COPD, focusing specifically on how patients decide for the right moment to start treatment with antibiotics and/or oral corticosteroids, what they consider important when making this decision and aspects which might interfere with successful implementation.

**Methods:**

We interviewed 19 patients with chronic obstructive pulmonary disease using qualitative semi-structured interviews, and applied thematic analysis for data analysis.

**Results:**

Patients were well equipped with experiential knowledge to recognize and promptly respond to worsening COPD symptoms. Worries regarding potential adverse effects of antibiotics and oral corticosteroids played an important role in the decision to start treatment and could result in hesitation to start treatment. Although self-treatment represented a practical and appreciated option for some patients with predictable symptom patterns and treatment effect, all patients favoured assistance from a medical professional when their perceived competence reached its limits. However, a feeling of obligation to succeed with self-treatment or distrust in their doctors or the health care system could keep patients from timely help seeking.

**Conclusion:**

COPD patients regard self-treatment of exacerbations with antibiotics and/or oral corticosteroids as a valuable alternative. How they engage in self-treatment depends on their concerns regarding the medications’ adverse effects as well as on their understanding of and preferences for self-treatment as a means of health care. Caregivers should address these perspectives in a collaborative approach when offering COPD patients the opportunity for self-treatment of exacerbations.

**Electronic supplementary material:**

The online version of this article (doi:10.1186/s12875-017-0582-8) contains supplementary material, which is available to authorized users.

## Background

Chronic obstructive pulmonary disease (COPD) is a leading cause of morbidity and mortality, associated with a considerable economic and social burden. Particularly acute exacerbations of COPD (AECOPD) stand for high hospitalization and mortality rates, health costs, accelerated progression of lung function decline and reduced health-related quality of life [1–3]. Since early treatment is important for faster recovery and for reducing the overall burden of exacerbations, delay in help seeking is of major concern [4, 5]. Providing patients with antibiotics and/or oral corticosteroids and education about prompt initiation of treatment when experiencing deteriorating symptoms, appears a reasonable strategy to handle this concern.

While such self-treatment is obviously effective for some patients [6–8], high non-adherence rates and even higher mortality rates in patients with self-treatment plans [9] have been reported. Moreover, even though both COPD patients and health care professionals seem positive about the concept of AECOPD self-treatment [10, 11], concerns have been raised in terms of choosing the appropriate patients, and the importance of education and ongoing communication has been emphasized [11, 12]. As self-treatment of AECOPD is often studied as part of comprehensive self-management programs, their explicit role within these programs is difficult to determine [13]. Moreover, beyond the knowledge that limited education is obviously not sufficient to turn COPD patients into effective self-treaters [13], it remains unclear whether self-treatment of AECOPD itself could compose an effective intervention in routine care and what is required to ensure successful implementation in everyday practice. Despite these gaps in knowledge, AECOPD self-treatment with antibiotics and/or oral corticosteroids is already recommended in some COPD guidelines [14, 15].

If we want to establish self-treatment of AECOPD as part of everyday, routine COPD care, it is important to gain understanding of the patients’ perspective, as this could help identify potential barriers to successful implementation. So far, existing studies on the patients’ perspective on AECOPD and self-treatment can only give limited insight into how COPD patients would actually make use of self-treatment plans. Underlying reasons for their self-treatment behaviour are little understood [11, 16–19]. This study of COPD patients’ experiences with, and perspectives on, AECOPD self-treatment aimed specifically at how patients decide on the right moment to start treatment with antibiotics and/or oral corticosteroids when experiencing worsening symptoms, what they consider important when making this decision and what aspects might interfere with the successful implementation of AECOPD self-treatment in routine care.

## Methods

### Methodology

The aim of the study required a qualitative study design suitable for acquiring information about participants’ experiences and meanings. We chose semi-structured in-depth interviews to get hold of the participants’ stories. We based our study on the assumption that behaviour in a situation of ill health is more than a reaction to physical symptoms resulting from a biological process; rather, we assume that it results from interpretative processes stimulated by social interaction through which persons give meaning to their experiences and social encounters [20].

### Sampling and materials

**Fig. 1:**
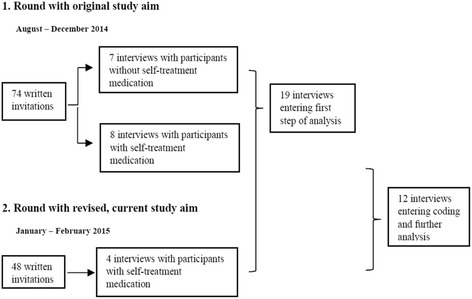
Flowchart showing the recruitment and sampling process

This study aimed at exploring the perspectives of COPD patients who have received antibiotics and/or oral corticosteroids as part of everyday, routine care, and not as part of comprehensive self-management programs. We recruited participants via the responsible physician at a heart-and-lung rehabilitation institution in northern Norway, who sent invitation letters to 122 COPD patients in two rounds (74 in the first round and 48 in the second round). They had taken part at least once in a 4-week lung rehabilitation program in 2012/2013, during which patients get information about AECOPD but are not systematically instructed in AECOPD self-treatment. We asked them to note on the consent form whether they had received any type of medication they should use when experiencing a worsening of their COPD. We received 45 signed consent forms and one email with written consent. Three invitation letters were returned because of an unknown address and two other invited participants had passed away. In a first round, we contacted 15 of the 46 respondents by phone, obtained oral consent and arranged a date and time for the interviews. This sampling was done purposefully to include participants from rural and urban areas, both sexes and with different working status. Originally, the study aimed at exploring self-treatment of AECOPD in a broader sense, including non-medical self-care activities, which is why we also included seven participants who had not received antibiotics and/or oral corticosteroids. These interviews added analytic insight on living with AECOPD and help seeking, but not so much on self-care activities and especially not on self-treatment with antibiotics and/or oral corticosteroids - as these seven participants had not received these. Therefore, after thorough reflection, we decided to narrow down the study aim to particularly explore self-treatment with antibiotics and/or oral corticosteroids, considering that in-depth knowledge about the self-treatment with antibiotics and/or oral corticosteroids presented itself as significant topics of concern, compared to broader, more general insights into COPD patients’ self-care behaviour. In other words, we decided to report mainly from a larger subsection of the data. We supplemented this new approach with another four interviews with respondents who had received antibiotics and/or oral corticosteroids (after the second round of invitation letters). After 19 interviews in total and intermediate analysis of these, we found we had obtained saturation [21] in terms of having a rich dataset to answer the altered research question without new topics emerging from further data collection. See Fig. 1 for an overview of the sampling and data collection process.

The interviews took place in participants’ homes (or in two cases at the University of Tromsø) and lasted between 60 and 120 min. All were conducted by the first author (a general practitioner trainee and PhD fellow), supervised by the last author between interviews, and followed an interview guide with the same key-questions in all interviews (see Additional file 1). Focus was on recognition of exacerbations, self-help activities, experiences with and reasons for using antibiotics and/or oral corticosteroids, attitudes towards being a “self-treating” person, and experiences and perspectives on seeking help from health professionals when experiencing a worsening of their COPD.

A carefully trained research assistant transcribed the interviews verbatim from audio recordings. The participants received a gift coupon of 500 Norwegian krone as compensation for participation.

### Data analysis

We employed thematic analysis [22] and an inductive approach for data analysis. Nvivo 10 data analysis software [23] and Mindjet MindManager Professional [24] were used as tools to organize the transcripts, codes, memos and relationships between them, and to visualize our findings. During data collection, interviews were read consecutively with an analytic view to emerging patterns, something which resulted in doing 4 more interviews than originally planned. In the first step of the actual thematic analysis, the 19 transcripts were read by the first and last author to obtain an overall impression. As a next step, the first author developed codes from the 12 interviews of those participants who had received antibiotics and/or oral corticosteroids. Due to the revised study aim, the remaining seven interviews were not coded or used for supporting the results section, but supplemented the analysis in terms of showing similar patterns on living with AECOPD and help seeking as the main 12 interviews (see Table 1). Coding was done on a semantic level, focusing on experiences and actions as well as related thoughts, attitudes and motives relevant for identifying recurrent patterns in the data. Codes were constantly verified by going back to the data. The last author reviewed the codes and related data extracts, and disagreements were clarified by discussion between the first and last author as well as by occasional re-coding during the analytic process. Subsequently, the first author examined relations between the codes in terms of motives, conditions and consequences of actions and looked for potential hierarchies to develop preliminary themes. These comprised patients’ experience from earlier exacerbations, concerns about medications and interaction with health care professionals. In order to minimize personal bias, moving from codes to preliminary and then to the final themes included constant reflections, literature reading and discussions with the other authors and peers. Moreover, themes were constantly tested by moving between the data, codes and visual theme maps to ensure that they represented the meanings found in the data and their explanatory power [17].

**Table 1:** Participant demographics

Participant	Sex	Age range	Civil status	Residence	FEV_1_/FVC
1^a^	male	55–59	married	urban	32%
2^a^	male	70–74	married	rural	98%
3^a^	female	75–79	widow	rural	34%
4^a^	female	70–74	married	rural	39%
5^a^	male	60–64	partner	urban	37%
6^a^	male	65–69	married	urban	45%
7^a^	female	60–64	divorced	rural	59%
8^a^	male	65–69	single	urban	63%
9	male	60–64	married	rural	n.a.
10	male	70–74	married	urban	47%
11	female	65–69	divorced	rural	39%
12	female	75–79	married	urban	91%
13	female	55–59	single	rural	84%
14	male	55–59	single	rural	n.a.
15	male	65–69	married	urban	45%
16^a^	female	70–74	married	urban	n.a.
17^a^	male	60–64	partner	urban	20%
18^a^	male	70–74	widow	rural	22%
19^a^	male	60–64	single	urban	64%

*FEV*_*1*_ */FVC* forced expiratory volume in one second/forced vital capacity, *n.a.* not available zzzz

^a^ participants who have received antibiotics and/or oral corticosteroids for AECOPD self-treatment and whose interviews were coded in detail

## Results

Our analysis revealed three overarching themes relevant to the participants’ decision-making about self-treatment with antibiotics and/or oral corticosteroids. *“Knowing their own body and illness”* describes the routine with which all participants used their personal experiential knowledge when assessing and responding to deteriorating symptoms. *“Negotiating the necessity of strong medications”* illuminates that deciding to start self-treatment requires, in addition to mere symptom recognition, an assessment of whether symptoms are serious enough to condone the risk related to potential adverse effects of the self-treatment medications. Finally, *“Experiencing the limitations of lay-medical competence”* gives insight into how differences in making use of the self-treatment medications and in help seeking is linked to aspects of participants’ earlier experiences with health care professionals and relationship to doctors.

### Knowing their own body and illness

Most of the participants did not remember when or how they obtained the self-treatment medications for the first time, or the instructions for using them. However, all seemed to have understood that they should take them when “getting worse”. Even though ‘getting worse’ was experienced differently, predominant symptoms to react upon were slowly increasing breathlessness and worsening general condition over several days, eventually accompanied by increased mucus production, more coughing or a feverish feeling.

*“…I recognize this lung illness as it comes with a lot of mucus, I start coughing and there is a lot of mucus and so on,…”*

*(woman, participant 4)*

As most participants had lived with COPD for several years and had experience from earlier episodes often occurring with similar symptom patterns, both emotional and practical responses to worsening symptoms seemed quite routine. Becoming sick often encompassed restrictions in daily life that could cause frustration. Depending on what was considered appropriate to relieve the symptoms, calming down, taking it slow, using specific breathing techniques, trying mucus-dissolving tablets or increasing the dosage of short-acting bronchodilators were applied as prompt self-help activities.

*“…but later on, I learned that, […], taught myself to calm down when I woke up and couldn’t breathe, get up and take it slow …*

*(man, participant 6)*

If symptoms clearly indicated more severe illness, or when these ‘first choice’ procedures failed, participants would think that stronger treatment, namely antibiotics and/or oral corticosteroids, could become necessary.

*“Now I feel it, now there’s something going on in my body […] something, now I start freezing, fever goes up until over 39, then there’s something that is not right, …”*

*(woman, participant 16)*

Both a vague feeling of embodied knowledge, but also more objective signs could indicate serious illness. Yet, participants had different perceptions of when a condition was serious enough, and of which feelings, signs and symptoms actually indicated the need for stronger treatment. Even though objective signs like fever or coloured sputum seemed to make the decision easier for some participants, we did not find an overall pattern in the data showing that the presence of objective signs was critical for making a straightforward decision.

*Interviewer: «When do you start with the treatment?»*

*Woman: “This exactly is a bit difficult, and I think it might also be difficult for you doctors. Yeah, because, am I sick enough? Is this a worsening or just a change in the weather? I am a bit sensitive for when the outside temperature goes up and down […]. So, am I sick enough? Or, can I manage the symptoms without the medications?*

*(woman, participant 11)*

Rather than regarding certain signs/symptoms as absolute indicators for starting treatment, the overall important question was whether these were serious enough relative to the medications’ potency and potential adverse effects.

### Negotiating the necessity of strong medications

The necessity for stronger medications was always associated with the wish to avoid certain consequences of becoming seriously ill, such as unwanted hospitalizations and extensive breathlessness and exhaustion both in the short- and long-term. The participants regarded antibiotics and/or oral corticosteroids as potent treatments to prevent these consequences.

*“I know my body best, don’t I? I know best that, well, now I am getting worse, and then it is just good to have them [the drugs] on standby, they have helped me so many times…”*

*(woman, participant 16)*

At the same time, many participants could have been warned of the risks associated with taking medications from their physicians, or could have heard rumours about the medications’ downsides. This lay-medical understanding of the medications’ effects could result in associations between the medications’ potency and adverse effects or risks due to inappropriate use.

*“No, it has to do with the fact that I know prednisolone makes blood vessels thinner, and, and this tells me that when blood vessels get thinner it can be much easier to maybe get a stroke or something, eh, and, you won’t go around eating this… when you know that there can be dangerous side effects of some kind, right?” (man, participant 6)*

Even though not all participants had similarly strong thoughts about the medications as this man, the perception of the self-treatment medications as ‘double-edged sword’, and the influence of both the ‘effect’ side and the ‘concern’ side on the decision to start treatment, emerged as a clear pattern across the dataset.

Positive and seemingly well-functioning AECOPD self-treatment experiences were connected with a certain self-confidence to interpret signs and symptoms correctly and a strong appreciation of the quick symptom relief. Disregarding concerns about the medications’ adverse effects alleviated the decision to start treatment as soon as the first signs of illness occurred. In contrast, participants being very sceptical towards drugs in general were more aware about potential treatment related risks. Certain signs and symptoms did not easily convince them about the necessity of the disliked medications. They had concerns regarding the use of oral corticosteroids and were afraid this might directly damage bodily structures. Antibiotics, they thought, had indirect adverse effects mostly on their own health, such as the diminishing effect in future exacerbations. Talking about antibiotics in a general way as below also indicated that the participants refer to a general public health narrative on antibiotics.

*“Yes, no, that’s the point, well, you’d wait a bit before starting this process, ‘cause it’s a kind of serious process, to start taking antibiotics.”*

*Interviewer:” Serious process?”*

*Man:” Well, yeah, it is kind of serious.”*

*Interviewer:” Why?”*

*Man:” eh, you, you shouldn’t abuse antibiotics. So, you’d wait an extra day to see how it goes before you start.”*

*(man, participant 1)*

Participants with such strong concerns could have a high threshold to start self-treatment with antibiotics and/or oral corticosteroids and seemed to wait with starting treatment until their condition got very serious in their opinion. Most participants, however, had a more moderate stance. Those without very strong concerns about the medications had more difficulties to weigh the necessity for treatment up against the risk of leaving their symptoms untreated. Particularly a combination of slowly developing signs and symptoms and considerable concerns about antibiotics and/or oral corticosteroids could cause insecurity regarding the necessity for treatment, eventually leading to a drawn out decision-making process. Yet, all participants seemed to have individually varying perceptions of the right moment to start treatment, depending on e.g. feelings of competency and attitudes towards help seeking.

### Experiencing the limitations of lay-medical competence

Despite the treatment related concerns, the participants shared a principal understanding that the idea of self-treatment could represent a practical alternative to potential difficulties with getting an emergency appointment and to the difficulties of traveling to the doctor’s surgery when feeling very ill. Moreover, having effective medications on ‘stand-by’ created an overall sense of security. However, their stories accounted for substantial differences in how they regarded themselves as ‘self-treating’ persons and how they made use of their self-treatment opportunity. These differences seemed closely connected to their prior experiences with receiving care from a health care professional, which is particularly evident in stories about when to consult a doctor for assistance.

*“… you feel that breathing is getting heavier and maybe feel under the weather, well, this could indeed mean that you have an infection in your body.”*

*Interviewer: “Mmm, and then? Do you think taking antibiotics or doxycycline yourself, is this…?”*

*Man: “No, I wouldn’t take it without having a doctor checking me first, I think this is how it’s supposed to be.”*

*(man, participant 5)*

This man’s reasoning illustrates an attitude held by a number of participants who had, with the exception of when being out of the country, never treated themselves without having consulted the doctor before. These participants seemed neither less able than others to interpret their symptoms, nor extremely concerned about the medications strengths. They just wanted a confirmation before starting treatment, as they allocated the expertise and decisional power about these medications to the doctor. However, this rather paternalistic understanding of the doctor-patient relationship did not impede their active and confident participation in managing ‘their’ illness. Rather, regarding the doctor’s expertise as supplement to their own experiential knowledge reflected a wish to collaborate with their physician for ensuring optimal treatment. In contrast, other participants had treated themselves once or several times in the past. When symptoms occurred as anticipated according to certain symptom patterns, treatment seemed to be effective and was apparently carried out with confidence. However, even the most confident ‘self-treater’ could experience treatment failure or insecurity whether treatment with antibiotics and/or oral corticosteroids was really necessary. In such cases, they reached the limits of their lay-medical competence and needed professional help.

*“…[if] I, over several days, do not feel and see [that the treatment helps], well, I can tell very well by the phlegm when it gets better, then it’s not olive green anymore. When it’s still olive green, then, then danger is really ahead. Yes, then one heads downtown [to the doctor], I promise you (laughs).” (woman, participant 11)*

Participants such as this woman, and those who would always consult a doctor before starting treatment, regarded help seeking as natural and basically unproblematic. Other participants, however, tended to hesitate with consulting a doctor when feeling insecure or in case of treatment failure. One underlying reason seemed a desire to ‘bypass’ an encounter with their doctors by clinging to the self-treatment opportunity.

*“Well, I wouldn’t call [call the doctor] without…thinking that I needed to go to the hospital to get some help. I’ve been down [very sick] so many times that, that, well, I’ve always got up on my feet again, but it’s, it takes often quite a long time to get better.”*

*(man, participant 18)*

Several participants who trusted their own knowledge more than, or at least as much as, the doctors’ expertise, shared this man’s attitude that doctors had not more help to offer than they could provide for themselves. Their stories revealed negative experiences of having received care for earlier AECOPD or other illnesses. They seemed to avoid contact with doctors whenever possible, even though this would mean a prolonged course of illness. Their confidence in self-treatment seemed to a considerable extent be based on distrust in the healthcare system. Other participants actually appreciated the reassuring care of a doctor when feeling insecure, but hesitated anyway with seeking help.

*“Well, I wish I could [call the doctor]. But it’s so deep inside me, that… I’m afraid to bother [the doctor], I don’t want to bother [them], you see?”*

*Interviewer: “Explain a bit more, will you?”*

*Woman: “Well, that I should call and call and call and bother him with the same things, well, I should know it, after so many years I should know it myself.”*

*(woman, participant 11)*

The reasoning behind their hesitation to seek help comprised worries of being burdensome to the doctor, feeling obliged to be able to manage ‘their illness’ on their own. Instead of regarding insecurity of making a distinct treatment decisions as a natural limitation of a layperson’s medical competence, they would perceive it as personal incapability or failure, eventually keeping them from timely help seeking.

## Discussion

### Main results

The present study aimed at exploring the use of AECOPD self-treatment with antibiotics and/or oral corticosteroids from the patients’ perspective, focusing on aspects relevant to their decision of when to start treatment and on potential barriers to more successful implementation of this care strategy. We found that the decision to start self-treatment required not only symptom recognition but also symptom assessment in terms of evaluating whether the illness was serious enough to condone the treatment related risks. This assessment could be easy when symptoms occurred according to the patients’ regularly experienced symptom patterns, and clearly indicated that treatment with antibiotics and/or oral corticosteroids was the best alternative for symptom relief. Yet, when signs and symptoms were ambiguous, or in case of treatment failure, assistance by a medical professional could be necessary. Help seeking, though, could be, but was not always natural to all participants. Distrust in receiving appropriate care, or feeling obliged to succeed with self-treatment could be barriers to timely help seeking when needed.

### Strengths and limitations

Our purposeful sample ensured that all the participants had a comparable level of education about COPD through the rehabilitation program. Perspectives from both sexes and different age groups, working status, residence and different sources of the self-treatment medications ensured considerable variation in the sample. This is a strength of our study, as it enables us to acquire insight into COPD patients’ real-life experiences, rather than depicting idealized self-treatment strategies as described in the literature and in policy statements. Yet, this has implications for the transferability of our findings. On the one hand, this study adds valuable knowledge to inform actors involved in implementation of AECOPD self-treatment into routine care, such as primary care physicians. On the other hand, transferability to COPD patients receiving more comprehensive instructions about self-treatment, as for instance in randomized self-treatment trials, might be limited. Another limitation is that participants may in some cases have not talked about AECOPD in a medical sense, but about illness episodes assumed to be exacerbations resulting in actions such as self-treatment or seeking help from health care professionals. Choosing another sampling strategy that ensured that participants had been diagnosed with AECOPD would perhaps have resulted in other findings. However, we considered our method more appropriate because self-treatment also includes self-diagnosis rather than diagnosis by a professional. Furthermore, we acknowledge that adjusting the study aim and subsequently, the approach to data collection, during the study period is debateable in terms of sampling bias. Yet, we regard our choice as appropriate according to our experiences and reflections during the data collection process.

### Discussion of the main results

Our findings that COPD patients have considerable experience-based knowledge and skills to recognize and promptly respond to symptom changes are in line with the results of previous studies [17, 19, 25, 26]. Self-treatment with antibiotics and/or oral corticosteroids may in fact compose a practical alternative to traditional care, particularly for patients with recurrent symptom patterns clearly indicating when treatment beyond increased dosage of maintenance treatment or non-medical self-help is necessary. ‘Knowing the patient‘, in terms of knowing their usual presentation during an exacerbation, seems therefore important to identify patients potentially benefiting from self-treatment [11, 27]. The importance of individualizing treatment plans is already widely recognized and implemented in self-treatment interventions [6, 28]. However, this disease-focused approach to self-treatment education does not consider the patients’ perception of and attitude towards utilizing ‘their’ plans.

One of our main findings was that the decision to start self-treatment with antibiotics and/or oral corticosteroids always included an evaluation of whether leaving the illness untreated composed a greater health risk than the potential side effects of the medication. Especially when symptoms were diffuse and not clearly indicated the need for treatment, we found that patients would hesitate with starting treatment or not start treatment at all by themselves. COPD patients’ concerns regarding the self-treatment medications appear in earlier studies, but are often not further discussed as a key to patients’ self-treatment decision-making [16, 17]. Yet, patients’ double-edged perspectives on drugs in general and their influence on medication taking behaviour has long been understood [29–35].

According to the Necessity-Concerns-Framework, for instance, treatment cognitions are equally important as illness cognitions to determine patients’ treatment-related decision-making. Its core concept comprises a “necessity-concern-dilemma”, which describes a patient’s judgement about the personal need for treatment as opposed to concerns about potential negative consequences [34]. Even though the Necessity-Concerns-Framework is an extension of Leventhal’s Common Sense Model of Illness Representations [36], which is regularly brought up in the COPD self-management literature, the framework remains to our knowledge unnoticed in the theoretical foundations of COPD self-management interventions [37]. Treatment cognitions are important in our study, yet, we do not know to what extent patients’ treatment related concerns in fact contribute to inappropriate treatment decisions in a medical sense, or whether patients with less concerns and clearer symptoms are those with better outcomes in self-treatment interventions.

Importantly, our results clearly show that patients can feel uncertain about symptoms and treatment necessity or may experience treatment failure. They might over-or underestimate the necessity for treatment with antibiotics and/or oral corticosteroids according to what is appropriate from a medical point of view. Therefore, as clinical examination and diagnostic tests can help to better target AECOPD treatment to the underlying cause, involving a health care professional in the assessment of AECOPD would at least make sense if symptoms are ambiguous. Yet, our findings suggest that patients’ decisions regarding self-treatment and help seeking are not motivated by pure medical aspects, but mostly by their previous encounters with doctors and experiences with the health care system. This raises questions regarding the ‘real’ impact of self-treatment interventions on patients’ behaviour. In theory, self-treatment education should increase patients’ self-efficacy in interpreting their symptoms, contribute to patient autonomy and to less need for health care contacts [37, 38]. Even though these goals may have partly been reached, the overall evidence remains inconclusive [38]. According to sociologically inspired investigations on health care utilization of people with chronic illnesses, it is clearly the recursive nature of health care contacts throughout a patient’s individual illness trajectory that determines current and future health care utilization patterns [39–41]. Neither changes in the delivery of health care nor the “expressed need” for help were found to predict a patient’s self-care and help seeking behaviour [40, 41]. This understanding is reflected in our participants’ help seeking attitudes. Participants principally contacting their doctor before starting treatment did not seem to have adopted self-treatment as a new form of AECOPD care even though their experiential and lay-medical knowledge was probably sufficient to negate the need for help in the first place. Moreover, we argue here that participants who in fact used their self-treatment medications did so because the self-treatment opportunity served to maintain their already existing pattern of interacting with the health care system. Importantly, the introduction of new means of health care, such as AECOPD self-treatment, might even threaten patients’ established relation to health care professionals and result in tensions unsupportive of the implementation of these new care forms [42]. We find that such a tension is reflected in participants who felt uneasy to contact their doctor due to the feeling that they should be able to manage AECOPD self-treatment on their own. Engaging in self-treatment to avoid contact with a doctor or due to worries of being burdensome would then, at least partly, derive from a dysfunctional patient-physician relationship instead of a patients’ medical confidence. On the one hand, this may raise concerns regarding the increased use of antibiotics and oral corticosteroids, which was found in patients in self-treatment intervention groups [43–45]. On the other hand, patients engaging in self-treatment or hesitating to contact the doctor would be consistent with other findings showing that a number of self-treatment interventions reduced the number of health care contacts [44–47].

The above discussion mirrors further the results from a study suggesting that self-care and help seeking patterns of people with chronic illnesses are “intrinsically intertwined” and result from a dynamic relationship between life-world experiences and experiences from illness trajectories [39]. This understanding implies that patients make appropriate and logical decisions according to their lay-medical understanding of illness and medications, and the impact of symptoms on their daily living. While COPD patients’ motivation to engage in self-treatment or to seek help might express their ‘voice of the lifeworld’, caregivers and policy makers may promote self-treatment with ‘the voice of medicine’ [48]. Listening closer to the patients’ voice, that is acknowledging their basic concerns, patterns of relationships to health care and experiences from everyday management of their illness, could help caregivers to identify discrepancies between their own and the patients’ motivations for AECOPD self-treatment [48, 49]. This could help to overcome potential barriers to the implementation of AECOPD self-treatment with antibiotics and/or oral corticosteroids in routine care, and would strengthen the understanding of COPD self-management, including AECOPD self-treatment, as one element of integrated care instead of regarding it as the ultimate goal in itself [50, 51].

## Conclusions

AECOPD self-treatment can be a valuable alternative to traditional care, especially for COPD patients with recurring AECOPD symptom patterns. Yet, to start treatment with antibiotics and/or oral corticosteroids is not a straightforward decision purely based on signs and symptoms. The patients take their previous experience into account and, like doctors, balance benefits and risks related to the potent medication. Moreover, patients’ attitudes and preferences regarding self-treatment and interaction with health care professionals are influencing patients’ decision to seek help when needed. We suggest that giving patients the responsibility to self-manage potentially severe illnesses such as AECOPD should be regarded as supplementary to care by a health care professional. Moreover, AECOPD self-treatment should follow a collaborative approach, comprising a medical and social dimension. That is, caregivers should consider patients’ experiential knowledge about their AECOPD symptoms patterns when choosing eligible patients for self-treatment (medical dimension). Moreover, when tailoring self-treatment plans for individual patients, caregivers should try to determine patients’ understanding of and preferences for self-treatment as a means of health care (social dimension), including thoughtful and respectful communication about medication-related concerns and potential barriers to help seeking. This could help practising clinicians to establish and maintain a collaborative relationship with their patients and to improve the implementation of AECOPD self-treatment in routine care.

## Additional file

Additional file 1: Interview guide (English version). The Norwegian version of this interview guide was used in all 19 interviews. The English version translated by the main author JL is available as additional file. The interviewer did not strictly follow the outline of the interview guide and did not necessarily ask the questions as they appear in the interview guide, but adapted the questions in every interview. The interviewer was mainly guided by the four main topics that can be found as underlined headings in the interview guide. (PDF 102 kb)

## Abbreviations

AECOPD: Acute exacerbation of chronic obstructive pulmonary disease
COPD: Chronic obstructive pulmonary disease
