# Supplementary material for: Self-treatment of acute exacerbations of chronic obstructive pulmonary disease requires more than symptom recognition – a qualitative study of COPD patients’ perspectives on self-treatment
Source: BMC Fam Pract. 2017 Jan 25;18:8. doi: 10.1186/s12875-017-0582-8 (PMC5264444; doi:10.1186/s12875-017-0582-8)
Supplement: Additional file 1: — Interview guide (English version). The Norwegian version of this interview guide was used in all 19 interviews. The English version translated by the main author JL is available as additional file. The interviewer did not strictly follow the outline of the interview guide and did not necessarily ask the questions as they appear in the interview guide, but adapted the questions in every interview. The interviewer was mainly guided by the four main topics that can be found as underlined headings in the interview guide. (PDF 102 kb) [file 12875_2017_582_MOESM1_ESM.pdf]

Additional file 1, Interview guide (English version)

- Can you tell me a little about yourself? What you are doing during the day? Where and who you live with and what you work with?
- When did you get the diagnosis COPD? What kind of treatment do you get? Do you have other illnesses you take medications for?

Patients' understanding of, reactions (emotional, cognitive) on and behavior during an exacerbation

- What does "COPD exacerbation" mean to you? How would you name worsenings?
- How do you know it is the COPD and not something else?
- What do you think and feel when getting a worsening?
- What is the worst about a worsening?
- How does a worsening influence your work/live/mood/social life?
- What do you do to handle a worsening?
- What and/or who helps you the most when you have a worsening?
- Who do you seek help from?
- What would be even more helpful to you when having a worsening?

Patients' attitude towards and experience with self-treatment

- Where/from whom did you get the medications?
- Why did you get the medications?
- Can you tell about the information you received on how to interpret symptoms and use the medications?
- What did you think about it?
- How did it feel to have medications for own use?
- What did the doctor tell you to do when having a worsening?
- When should you use the medications?
- Did you receive written instructions? May I see them?
- Do you think they are easy to understand?
- Can you tell me about a situation when you used the medications?
- Was it difficult to know whether you should take them?
- If never used: have you ever considered taking them?
- What made you take the medications/not take the medications, and in this case, what did you do instead?
- Would you recommend to a friend to have medications to own use?
- Did the medications help?
- What did you do/would you do if you would not get better from the medications?

Influence on the experience with the healthcare system

- Can you tell me about a situation when you contacted a doctor when your symptoms worsened?
- What was the reason for you contacting the doctor?

- What does the doctor do when you have an appointment for COPD?
- Do you think the doctor does a good job?
- Do you trust your doctor?
- What is different when you are treated in the hospital?
- Whom do you prefer to contact when you have a worsening?
- What can the family physician/hospital doctor help you with?
- Do you have less need to contact your doctor now?
- Is there anything different now regarding making an appointment?
- How important is the doctor for you in terms of treatment for worsenings?
- Did the treatment change, and in what way, after you got the self-treatment medications?
- If you wished your family physician to change something in the way he treats you, what would that be?

Influence on coping, responsibility, autonomy, safety

- What are the advantages/disadvantages with treating worsenings at home?
- Does this make living with COPD easier, and if yes in what way? If not, why not?
- Do you have more control over your COPD?
- What about the responsibility for treatment?
- How is it to make the treatment decisions yourself?
- Do you have other persons around you that can help you?

Is there anything else you want to talk about?
